# Immersive Virtual Reality for the Reduction of State Anxiety in Clinical Interview Exams: Prospective Cohort Study

**DOI:** 10.2196/18313

**Published:** 2020-07-09

**Authors:** Brendan Joseph Concannon, Shaniff Esmail, Mary Roduta Roberts

**Affiliations:** 1 Faculty of Rehabilitation Medicine University of Alberta Edmonton, AB Canada; 2 Department of Occupational Therapy University of Alberta Edmonton, AB Canada

**Keywords:** virtual reality, VR, head-mounted display, HMD, immersive technology, occupational therapy, OSCE, simulation, psychology, anxiety

## Abstract

**Background:**

Immersive virtual reality (VR) with head-mounted display was used to determine if clinical interview simulation could reduce the anxiety levels of first-year occupational therapy (OT) students as they prepared for upcoming Objective Structured Clinical Examinations (OSCEs). Anxiety among health science students is a potential problem that may diminish their performance during OSCEs. This investigation aimed to fill the gap in the literature regarding the effectiveness of VR to reduce anxiety in OT students.

**Objective:**

This investigation aimed to uncover the effectiveness of immersive VR in reducing state anxiety in OT students who were preparing for OSCEs.

**Methods:**

A prospective, experimental, nonrandomized controlled trial compared levels of state anxiety, test anxiety, and academic self-efficacy in two groups of first-year OT students; these levels were measured at four different time points by self-reported psychometric scales, analyzed with a mixed factorial analysis of variance (ANOVA). Members of Phase 1 (NoVR) were not exposed to the VR simulation and acted as a control group for members of Phase 2 (YesVR), who were exposed to the VR simulation. VR simulation featured a virtual clinic and a standardized patient who students could interview in natural language. Measures of student study strategies and previous experience with VR were also recorded.

**Results:**

A total of 49 participants—29 in the NoVR group and 20 in the YesVR group—showed that state anxiety had a rise-then-fall trend, peaking at the time point just before the OSCE. At that point, the YesVR students showed significantly less state anxiety than did the NoVR students (*t*_46.19_=2.34, *P*=.02, Cohen *d*=0.65, *ηp_2_*=0.105). The mean difference was 6.78 units (95% CI 0.96-12.61). In similar trends for both groups, student test anxiety remained relatively static across the time points, while academic self-efficacy continually increased. A moderate positive correlation was found for total time spent studying and peak state anxiety (NoVR *r*=.46, n=28, *P*=.01; YesVR *r*=.52, n=19, *P*=.02).

**Conclusions:**

This investigation shows evidence of immersive VR’s capability to reduce state anxiety in OT students preparing for clinical practical exams. Immersive VR simulation, used for the reduction of anxiety in health science students, can potentially lead to a future of positive mental health change from the virtual to the real world.

## Introduction

### Background

This investigation used immersive virtual reality (VR) to reduce anxiety among occupational therapy (OT) students who were preparing for a clinical practical exam. VR is a useful tool that can positively shape mental health. It utilizes a human-machine interface that immerses people into digitally rendered illusions that are multisensory in composition and projected by computer hardware. These illusions act as virtual environments, allowing people to condition themselves against symptoms of anxiety by undergoing VR exposure therapy (VRET), a form of systematic desensitization that facilitates mental fortification against a feared stimulus [1]. VRET allows for the training of affective regulation, while people are subjected to situational contexts that induce anxiety [1,2]. VRET can safely provide answers to inaccessible and intangible concepts by observing the responses of people who are subjected to fear- and anxiety-inducing stimuli, which would otherwise be considered too dangerous or unethical to perform in the real world [3]. Depending on the extent of a virtual system’s designed capability, a person immersed within the virtual environment acts as a user who may encounter, interact, control, and modify the virtual world. The user’s experience is *evoked* to improve their mental proficiency and habituate against fear and anxiety [1]. In this investigation, the anxiety under analysis is of the type that students may experience while preparing for clinical practical exams in health science programs.

### Campus Anxiety: A Prevalent Problem

Anxiety is a feeling of uneasiness and worry, usually generalized and unfocused as an overreaction to a situation that is subjectively seen as menacing [4]. It is a theoretical construct, capable of being triggered in either general or specific situations, with *proneness* (ie, trait anxiety) representing the frequency and/or intensity of the response and *transitory* (ie, state anxiety) representing the momentary response at a specific point in time [5]. Trait anxiety is a stable construct that is associated with personality traits, influencing the degree to which a person’s state anxiety response occurs within a specific point in time [5]. Spielberger and colleagues, in 1972 and 1978, developed a measure for test anxiety, which detects differences in test-specific personality traits between individuals.

Test anxiety is situation specific and associated with two components: (1) cognitive components that manifest symptoms of worry, due to student concerns regarding the outcome of an assessment, and task-irrelevant thinking, causing interference and shifting of attention to irrelevant content, and (2) affective components that manifest physiological reactions, such as increased heart rate and headache, nervousness, and tension (ie, emotionality) [6]. Self-centered worry cognitions and emotionality responses, which students may experience during testing situations, are potentially distracting and may disrupt concentration and attention, resulting in reduced performance on cognitive-intellectual tasks [7].

It is important to note that anxiety while under academic evaluation (ie, test anxiety) is normal, especially in situations where students have invested urgent and preparatory activities to win an ideal outcome. However, severe anxiety that causes students to “lock up,” panic, or show an unexpected reduction in performance is a serious problem. Anxiety symptoms are expected to have a negative impact on student academic achievement, self-efficacy, and self-concept [8]. In a survey with 1099 responses from a Canadian university, 38.5% of the university students self-reported that they had suffered from test anxiety at some point during their studies, 20.5% of the surveyed students believed that professors were unable or unwilling to help, and 11.3% of the students indicated they would not seek help as this would act against social desirability [9]. Test anxiety on university campuses is associated with student burnout and increased rates of attrition [10].

Self-efficacy is the subjective belief in one’s ability to successfully perform a given task [11]. Academic self-efficacy is of a specific type that pertains to academic situations, with greater levels being correlated with increased student class participation and exam performance at the higher grade-point average levels [12]. The relationship between academic self-efficacy and student anxiety, where the retention of academic self-efficacy is maintained by the suppression of state anxiety, was a primary outcome of interest in this investigation.

### VR Versus Anxiety

VR is defined as a human-machine interface that allows users to *project* themselves into a computer-generated virtual environment, where specific objectives can be achieved [13]. A potential method for reducing anxiety involves the use of immersive VR, which allows people to learn how they would feel and respond—physiologically, tactfully, and procedurally—while interacting with virtual situations that the brain treats as real. Immersive VR can change a user’s fear structure into an adapted one, removing the pathological kind that distorts reality and increases escapist tendencies [14]. The objective is to create an immersive virtual environment that simulates a specific testing situation, allowing users to learn how to adapt. This objective allows users the ability to develop anxiety tolerance by facilitating *inhibitory learning*, at both voluntary and involuntary levels, granting them resiliency after developing habituation from specific virtual situations to utilize in real-world situations [15]. *Inhibitory learning* is theorized to occur when anxiety suppression is achieved by neurobiological conditioning of the prefrontal motor cortex, amygdala, and hippocampus within the brain [16].

A fully immersive virtual environment allows users to accept and respond to artificial stimuli in a natural manner [13]. The component of VR that determines a user’s perception of their surrounding virtual environment is their physical level of *immersion*, ranging from nonimmersive (eg, desktop computer showing the environment) to fully immersive (eg, head-mounted display VR) [17-19]. Interactivity—the degree to which a user’s actions result in applicable responses within the virtual environment—is the second component of VR [19]. The third component is imagination: the degree to which a user feels he or she is within the virtual environment [19]. Presence is a subjective concept that defines the psychological degree to which a user understands where it is possible to act within the virtual environment [19]. People may feel deeply present in virtual environments when the experience makes them feel *involved* as they put their full attention on the virtual objectives [19].

These components influence VR’s level of fidelity, which is the capability of a virtual environment to reflect the real world. High fidelity is achieved when a user’s actions, senses, and thought processes in a virtual world closely or exactly resemble what would be transferrable to a similar situation in the real world.

In Gaggioli and colleagues’ report on the use of VR to reduce workplace stress for teachers and nurses, VR was found to be more effective in treating anxiety than the traditionally accepted gold standard for psychological stress treatment, cognitive behavioral therapy [20]. Sports psychologists have developed immersive VR environments that train an athlete’s mental concentration for sprinting events, depicting crowd-filled stadiums and competitors [21]. Designs of virtual hospital waiting rooms allow older adults the opportunity to be treated against anxiety-inducing stimuli, such as loud noises from distressed patients or crying infants [3]. This exposure could be employed to improve the efficacy of psychosocial therapy, such as cognitive behavioral therapy, with VR simulations resembling anxiety-inducing situations [3]. Kniffin and colleagues reported on diaphragmatic-breathing training for the retention of attentional control to enhance self-regulatory skills, which was tested on female students who were exposed to virtual avatars of aggressive males [2]. It was concluded that immersive VR was effective for the training of self-regulatory skills in this manner [2].

### VR in Health Science

OT has recognized VR as a potential tool for treating clients, including those diagnosed with stroke, hemiparesis, musculoskeletal injury, brain injury, cerebral palsy, neurodevelopment disorders, geriatric limitation, mental health, and complex or chronic pain [22]. However, reports of VR’s role in curricula for interprofessional skills training in students are typically peripheral in comparison [23]. OT will often employ Objective Structured Clinical Examinations (OSCEs), which are clinical practical exams that assess student core competencies, including procedural, clinical encounter, and history-taking skills [24]. OSCEs often feature standardized patients, who are actors trained to portray the characteristics of patients, giving students the opportunity to demonstrate their technical and nontechnical skills while in a controlled environment.

There are reports detailing the use of virtual standardized patients in medical education, allowing students to practice history-taking skills with reasonable differential diagnosis results [25]. However, there is a gap in the literature regarding the use of immersive VR systems for the reduction of anxiety in OT students. OT students are often expected to interview standardized patients during OSCEs while under formal evaluation, resulting in them potentially experiencing increased levels of state anxiety.

It is expected that an immersive VR simulation of a clinical practical exam will facilitate *inhibitory learning* in OT students, resulting in the suppression of their anxiety symptoms. These anxiety symptoms, which may impact student performance on clinical practical exams, are expected to be conditioned by immersive VR simulation, resulting in a reduction of student state anxiety levels and retention of academic self-efficacy levels. Immersive VR in this investigation is expected to demonstrate these positive changes in OT students and fill the gap in the literature regarding these conditions. The results of this investigation may inform future decisions of educational disciplines, considering the implementation of immersive VR for the reduction of performance anxiety, associated with clinical practical exams.

### Aim of This Investigation

This investigation aimed to uncover immersive VR’s effectiveness for reducing anxiety in OT students who were preparing for an OSCE. The human-machine interface utilized a head-mounted display to achieve an immersive experience, complete with speech-recognition software, allowing the use of natural language for conversing with a virtual standardized patient. This system was expected to optimize *inhibitory learning* for the facilitation of anxiety tolerance as detailed by Craske and colleagues’ report [15]. Academic self-efficacy was also measured to determine its relationship with peak state anxiety.

### Research Questions

This investigation was designed to answer the following research questions:

1. Does immersive VR simulation of a clinical practical exam (ie, OSCE) effectively reduce state anxiety in OT students when compared to a control group?
2. How is academic self-efficacy influenced by exposure to a VR simulation of an OSCE?

### Expectations

This investigation was expected to reveal the following:

1. If the VR simulation is effective as a form of VRET, we predict a reduction in state anxiety scores at times when the VR simulation is available.
2. If the OSCE is an anxiety-inducing event, we predict a peak in state anxiety scores at the time closest to the OSCE. However, we also predict that students who are exposed to the VR simulation will show lower peak anxiety scores than controls.
3. We predict an inverse relationship between measures of state anxiety and academic self-efficacy.

## Methods

### Experimental Design

This investigation was a prospective, experimental, nonrandomized controlled trial, involving two groups of participants, each comprised of OT students in the first year of their program. Members of Phase 1 (NoVR) were not exposed to the VR simulation and acted as a control group for members of Phase 2 (YesVR), who were recruited in the following year and were exposed to the VR simulation. The OT program itself was consistent in terms of faculty practice opportunities. Facility infrastructure, teaching of the OSCE content, sequencing of the courses, scheduling of mandatory practice sessions, and the professors themselves remained the same between groups.

Unlike a blinded randomized design, this investigation allowed each group of participants to be aware of their status in the experimental process. Due to the critical opportunities for when the OSCEs became available, it was not possible for this investigation to feature a block-controlled trial. Had a standard randomized controlled trial been utilized, there would have been difficulties with randomizing the students to their designated conditions; withholding an intervention that has the potential to positively impact student performance and well-being is unethical. This investigation used a wait-list control design that allowed participants from the NoVR group to access the VR simulation at a future schedule, separate from the YesVR group.

By maintaining intact cohorts as separate control and intervention groups between the years, this acted as a strategy to reduce treatment diffusion, which may have occurred if both groups had been analyzed at the same time. A potential confounding factor between the groups involves the time spent on opportunities to practice for the OSCE, regardless of modality. It was considered that the YesVR group’s state anxiety scores could have been influenced by additional time practicing with the VR simulation, regardless of its effectiveness. Therefore, it was important for both groups to log their total time spent in preparation for the OSCE to note any potential differences in time spent between groups.

### Recruitment

Announcements providing details of the investigation were made by an announcer who was neutral to the investigation’s outcome. The announcer was the same for each phase and was not a professor within the faculty; this was to minimize the compulsory pressure on students to participate. Announcements were made during a lecture to an OT class of 120 students for each phase. All OT students for each phase, who were in the first year of their program, were invited and eligible to participate. While students in both phases were informed of the availability of a survey package that became available for them to obtain and complete, Phase 2’s announcement included additional information, explaining the risks associated with the use of immersive VR hardware.

### Ethics

This investigation was approved by the Research Ethics Office of Research and Innovation, University of Alberta, Canada. After inspection, this investigation was deemed ineligible to record participant age and sex variables. This was to ensure participant anxiety scores would not be traceable by professors of the faculty, especially if that data belonged to participants who were unique to the student population and these participants could risk being identified. Census data pertaining to the demographics of the student body were allowable and have been provided in the results.

### Experimental Process

Students were requested to obtain and complete a survey package that contained four separate sections, each to be completed at different time points (TPs): TP1, TP2, TP3, and TP4. Each section contained questionnaires that recorded primary and secondary outcome measures of this investigation. Once each section was complete, the participants were instructed to drop off each section at a secure mailbox as indicated within the package information guide. Note that Phase 2’s (YesVR) package contained additional information about sign-up timeslots for immersive VR sessions, which would become available 2 weeks prior to their OSCE date. Tutorials on how to operate the VR hardware were provided by assistants, who were neutral to the investigation outcome and remained on standby at each appointed sign-up session. Each package section had been labelled with a specific completion date as follows:

1. TP1: 3 weeks before the OSCE.
2. VR sign-up became available for Phase 2 (YesVR) students only: 2 weeks before the OSCE.
3. TP2: 1 week before the OSCE.
4. TP3: 1 week after the OSCE.
5. TP4: 1 month after the OSCE.

**Figure 1:**
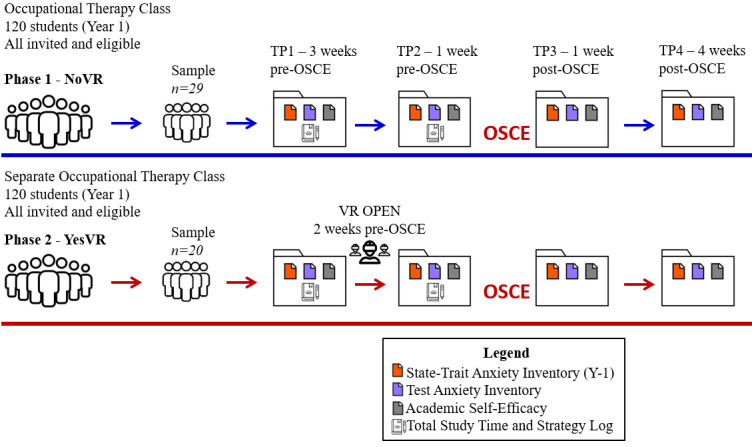
Experimental process of this investigation. NoVR: subjects not exposed to the virtual reality simulation; OSCE: Objective Structured Clinical Examination; TP: time point; VR: virtual reality; YesVR: subjects exposed to the virtual reality simulation.

Refer to Figure 1 for a summary of this investigation’s experimental process.

### Phases 1 and 2: Primary Outcome Measures

#### State-Trait Anxiety Inventory Forms

The State-Trait Anxiety Inventory (STAI) consists of two scales, each comprised of 20 items that measure anxiety in adults; they are scored as a value ranging from 20 to 80, with higher scores being associated with stronger symptoms of anxiety [5]. This investigation utilized STAI Form Y-1—the State Anxiety (S-Anxiety) scale—which measures a participant’s level of anxiety at a specific moment in time. The S-Anxiety scale has been found to be a “sensitive indicator of changes in transitory anxiety” as experienced by students exposed to stressors, such as job interviews or important school tests [5]. The STAI S-Anxiety scale was developed for use with college students and has shown a reliability stability of *r*<.62. Although reliability coefficients for the STAI have shown low-to-moderate scores, these stability coefficients are assumed for a state anxiety scale of this type, due to its expected ability to reflect differences in participant anxiety levels that are unique between each retesting situation [5]. Spielberger and colleagues [5] reported that normative Cronbach α coefficients for college students were .91 and .93 for males and females, respectively. For validity, the STAI S-Anxiety scale has been compared to other existing measures of state and trait anxiety in addition to contrasted groups, personality and adjustment tests, correlations with measures of academic aptitude, achievement, and investigations of the effects of different amounts and types of stress on S-Anxiety scores [5]. For college students, the Institute of Personality and Ability Testing (IPAT) Anxiety scale was compared to the STAI and showed validity correlation coefficients of *r*=.75 and *r*=.76 for females and males, respectively; however, a comparison between the STAI and the Taylor Manifest Anxiety Scale (TMAS) showed validity correlation coefficients of *r*=.80 and *r*=.79 for females and males, respectively [5]. The STAI has shown consistency in measuring essential qualities of anxiety, including apprehension, tension, nervousness, and worry [5]. The STAI Form Y-2—the Trait Anxiety (T-Anxiety) scale—measures a participant’s general and long-standing level of anxiety, which was not featured in this investigation. Overall, the STAI has shown to be both a reliable and valid instrument for measuring state anxiety levels in college students.

#### Test Anxiety Inventory

The Test Anxiety Inventory, also known as the Test Attitude Inventory (TAI), is a self-reporting psychometric scale that measures individual differences in test anxiety as a situation-specific personality trait. It is comprised of 20 items that measure anxiety attributable to test situations and scored as a value ranging from 20 to 80, with higher scores being associated with stronger symptoms. TAI subscales include worry and emotionality as major qualities of test anxiety [26]. Although most normative data for TAI usage is based on general-purpose or multiple-choice tests, it allows for modification about specific tests or time periods accordingly [26]. The TAI is also useful as a measure of outcome for studies featuring test anxiety treatment [27-29]. The TAI scale was developed for use with college and graduate students and has shown a reliability stability of *r*=.80 for time periods varying between 2 weeks and 6 months [26]. Spielberger and colleagues [26] reported that the TAI Total scale showed uniformly high scores for both males and females (.92 or higher), with median α values for worry and emotionality subscales of .88 and .90, respectively. For validity, the TAI scale has been compared to other existing measures of test anxiety, including the Test Anxiety Scale (TAS) and the Worry Emotionality Questionnaire (WEQ) [26]. For college students, the TAS and TAI comparison showed validity correlation coefficients of *r*=.83 and *r*=.82 for females and males, respectively; however, the TAI and WEQ-Emotionality comparison showed validity correlation coefficients of *r*=.85 and *r*=.77 for females and males, respectively [26]. Although there have been moderate positive correlations found when comparing the TAI with the STAI (*r*=.67 in males and *r*=.34 in females), the TAI was concluded not to measure or be comparable to state anxiety [26]. Overall, the TAI has been shown to be both a reliable and valid instrument for measuring test anxiety levels in college students.

#### Academic Self-Efficacy

In this investigation, academic self-efficacy was measured with the German Academic Self-Efficacy Scale (ASE), developed by Jerusalem and Satow in 1999 [30] as part of an extensive test battery to implement self-efficacy theory in schools of various grade levels up to and including trade school. Their instrument was developed by a combination of empirically proven concepts as outlined by Albert Bandura’s Self-Efficacy Theory [11,31] and Jerusalem, Mittage, and Satow’s research [30]. Their academic self-efficacy instrument is comprised of 7 items and showed internal consistency (ie, Cronbach α) of .73 when compared with the other tests that measured theoretically related constructs, such as optimism, helplessness, and social requirement expectations [32]. These theoretically related constructs were and were not related to academic self-efficacy (*r* values ranged from .27 to .51), resulting in theoretical correlations speaking for the criterion-oriented validity of the scale [30,32].

### Phases 1 and 2: Secondary Outcome Measures

Each survey package contained a log template providing instructions on how to note study activities and durations in preparation for the OSCE. Participants were encouraged by the instructions to log each study activity and its duration on an ongoing basis. Participants were requested to provide only the times and activities that were outside their normal class and lecture sessions. Phase 2 (YesVR) participants were also requested to include their VR simulation session in their log and, if applicable, provide special notes as to why their VR session was incomplete had it ended prematurely. In addition, detailed instructions to sign up for optional interviews and focus groups were provided in the packages. Interviews and focus groups took place both before and after the OSCE, with the goal of determining student viewpoints on requirements for simulation effectiveness, immersiveness, feedback, and improvement, as well as mental mindset before and after the OSCE.

### Phase 2 (YesVR) Only: Secondary Outcome Measures

A brief 5-item survey was an additional document available in the Phase 2 (YesVR) survey package, which allowed participants to define the amount of familiarity and ownership, if applicable, of immersive VR hardware they had experienced prior to the simulation as featured in this investigation. This survey established participant opinions regarding the following characteristics of VR environments: (1) VR features that they perceived to be the most important for establishing feelings of realism, (2) their preferred activities while using immersive VR, and (3) their prediction of immersive VR’s potential as an educational tool for the future of education. It was important for the survey to specify the type of VR in each question and provide examples of VR headsets—Oculus Rift (Facebook Technologies), Vive (HTC Corporation), PlayStation VR (Sony Interactive Entertainment), Gear VR (Samsung Electronics Co), or Google Cardboard—so that any potential discrepancy between the interpretation of immersive and nonimmersive VR types was minimized. A copy of this survey is available in Multimedia Appendix 1. Overall, this survey was used to establish a baseline understanding of participants’ attitudes toward immersive VR, prior to their involvement in the simulation as featured in this investigation.

### Simulation Design

#### Overview

The simulation in this investigation included the following components:

1. A virtual environment depicting a health sciences clinic, rendered with Unity game engine software (Unity Technologies).
2. Two virtual avatars: the first was a virtual standardized patient who was located within the virtual environment and would respond to a user’s questions; the second was a virtual exam evaluator who observed the user and would write notes into a clipboard during the interview process.
3. Speech-recognition software provided by IBM Watson, a question-answering engine linked with the virtual standardized patient.
4. VR (HTC Vive) and computer hardware that ran the software, allowing users to operate within the virtual environment itself.

#### Health Sciences Clinic

This project was designed and developed by an interdisciplinary team with experience in the use of VR learning objects for educational measurement, clinical evaluation, curriculum development, and assessment of student stress and anxiety. The project team members’ expertise and their associated departments included the following: computing science, physical therapy, communication and science disorders, rehabilitation medicine, and OT. Throughout simulation development, multiple demos were performed to allow revisions, based on user feedback from each session.

**Figure 2:**
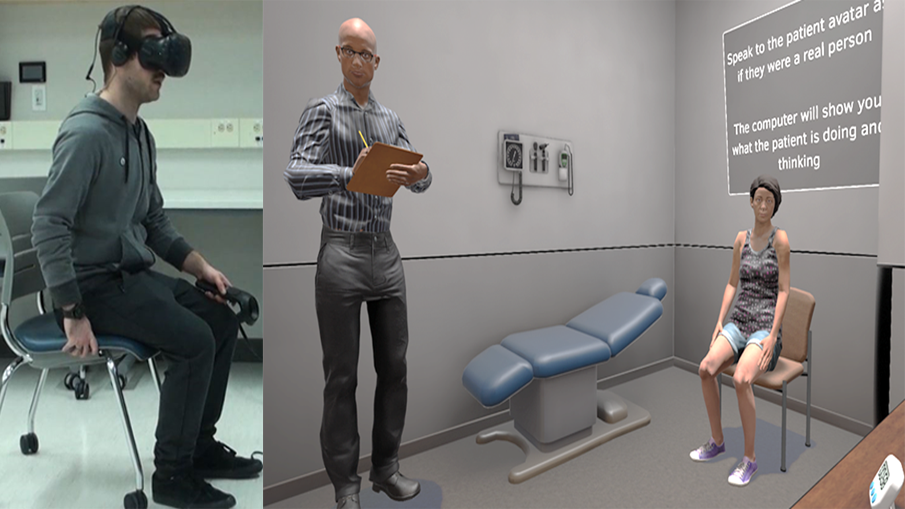
Screenshot of the virtual health sciences clinic.

Experts from the discipline of computer science were given a tour of the real-world health sciences clinic, allowing them to develop a virtual environment that closely resembled the OSCE setting as accurately as possible. The virtual environment was rendered with Unity game engine software and had two rooms: a hallway and an examination room (ie, doctor’s office) that were separated by a door. The setting allowed users to move through the hallway, open the door, and walk into the doctor’s office to meet the virtual standardized patient. The doctor’s office included a patient examination table and a computer desk that was outfitted with a desktop computer and a miniature clock. At this point, a buzzer was sounded to signal the start of the OSCE and the miniature clock began to count down from 8 minutes. The avatar representing the exam evaluator was standing discretely in the corner of the room, writing notes into a clipboard throughout the interview process. The avatar representing the standardized patient was sitting in a chair, next to the patient examination table. Both avatars were programmed to maintain eye contact with the user. Refer to Figure 2 for a sample screenshot of the virtual health sciences clinic.

OSCEs are described as follows, according to the Medical Council of Canada [33]: OSCEs are typically station oriented, attempting to resemble clinical scenarios with as much realism as possible. They are controlled and often feature trained actors who portray specific clinical patients in health-related situations. Although OSCE stations assess a variety of clinical competencies in students, assessments often focus on a student’s ability to communicate with the patient, typically in an interview process with a history-taking approach. OSCE stations are timed and formally observed by evaluators who assess the student’s performance.

#### Virtual Standardized Patient

The avatar representing the standardized patient was modelled to act as one of three different patients: Alex, Sam, or Jordan. They each had a different cause for their physical injury. The user could select a specific virtual patient or have one assigned randomly. They were voiced by the same voice actress (ie, standardized patient experience) and could respond to user questions or commands that were recognized and processed by IBM Watson’s voice-recognition software. The avatar would raise her arms above her head when asked to do so, having a noticeable reduction in her range of motion for whichever limb was injured. She would respond in a respective manner to other physical actions, such as when she was asked to reach behind her back or touch her head.

#### Speech-Recognition Software

IBM Watson was linked to Unity, via an application programming interface, with a script that contained programming code to access the microphone located on the VR headset. The script then streamed audio data to the Watson speech-to-text service, allowing the virtual standardized patient to convert the verbal question of a user to text and check it with a list of applicable responses. If a user’s verbal question matched an applicable response, the avatar would respond with an answer as previously voice-acted during her development. Her responses would vary depending on if she was Alex, Sam, or Jordan. Overall, the avatar was programmed to respond to an array of hard-coded questions that were divergent across the six components of health, including the physical, social, environmental, emotional, spiritual, and intellectual domains. She would also respond to other medical history questions, such as the reasoning of her doctor’s referral or whether she was prescribed medication. She would respond appropriately when greeted. She would state that she did not understand a question when a user issued a verbal command that did not match any line of text from the list of applicable responses. Note that she was not programmed to understand or respond to convergent questions, such as “Can you tell me more?”

Participants were informed to reword their question or change the topic entirely if the virtual patient repeatedly failed to understand a question. A list of questions that Alex, Sam, or Jordan could understand and respond to is available in Multimedia Appendix 2.

#### VR and Computer Hardware

This investigation’s VR hardware consisted of the HTC Vive, a consumer headset model with a built-in microphone, which allowed participants to interact within the virtual, health sciences clinic and converse with virtual standardized patients. The headset was supplemented with noise-cancellation headphones to reduce any real-world noise that could potentially contaminate the virtual clinic experience. The computer hardware was built using an Intel Core i5-6500K, 3.20 GHz CPU (central processing unit), NVIDIA GeForce GTX 1080 8 GB GPU (graphics processing unit), and 16 GB RAM.

### Statistical Analysis

A 2 × 4 mixed factorial analysis of variance (ANOVA) was used to evaluate differences between and within the scores of each phase’s STAI, TAI, and ASE. The two independent variables (ie, factors) were each designated with the following levels: 2 levels for representing each phase (ie, NoVR and YesVR) and 4 levels for representing each time point (ie, TP1, TP2, TP3, and TP4). Repeated-measures variables were corrected with Bonferroni *t* tests. Statistical significance was evaluated at α=.05, and a two-sided *P* value of .05 or less was considered to be statistically significant. Partial *η*^2^ (*ηp*^2^) effect size was checked to determine the ratio of variance accounted for by each effect and that effect plus its associated error variance within this ANOVA investigation. A *ηp*^2^ effect was considered meaningful if found to be 0.06 or greater, indicating the effect explained 6% of the variance in the dependent variable. Protected *t* tests were used to compare each specific time point between phases as well as the total time spent preparing for the OSCE between phases. The Cohen *d* effect size was checked for protected *t* tests between phases. To account for conceivable events where immersive VR may have shown results that were opposite in direction to the expected results, such as state anxiety being increased in students due to the VR intervention itself, analysis checks for differences were two-tailed. Pearson correlation coefficients were performed between both phases’ peak anxiety time points and study times, plus total peak anxiety and total academic self-efficacy. The peak anxiety time point was expected to be TP2, as it had the closest temporal distance to the OSCE of 1 week.

### Power Analysis

A power analysis was calculated using G*Power (Heinrich-Heine-Universität) [34] to determine the total sample size needed for each ANOVA—2 groups, 4 measurements, repeated measures, and between factors—and its associated scale (ie, STAI, TAI, and ASE). A power analysis of 0.8 with α=.05, expecting a large effect size for an ANOVA (*f*=0.40), required a total sample size of 34, with 17 participants required per group. Each ANOVA score result was considered meaningful if both the sample size and effect size thresholds were met (ie, 17 participants per group and *f*≥0.40, respectively).

## Results

### Overview

A total of 49 OT students participated in the study: 29 for Phase 1 (NoVR) and 20 for Phase 2 (YesVR). Although the response rate was 100% for package submissions, Phase 2 (YesVR) had 1 participant out of 20 (5%) fail to submit a completed TAI survey. Only 1 participant out of 20 (5%) from Phase 2 (YesVR) reported to have suffered from simulation sickness, yet was still able to complete the simulation. The majority of students in Phase 2 utilized the VR simulation for a single 15-minute session, with some having multiple sessions, which resulted in a mean VR simulation time of 17.32 minutes (SD 7.52) per student. Although this investigation was unable, ethically, to obtain participant demographics, census data from the OT student body are available in Table 1. The main statistical analysis results are provided in Table 2.

### State Anxiety

**Figure 3:**
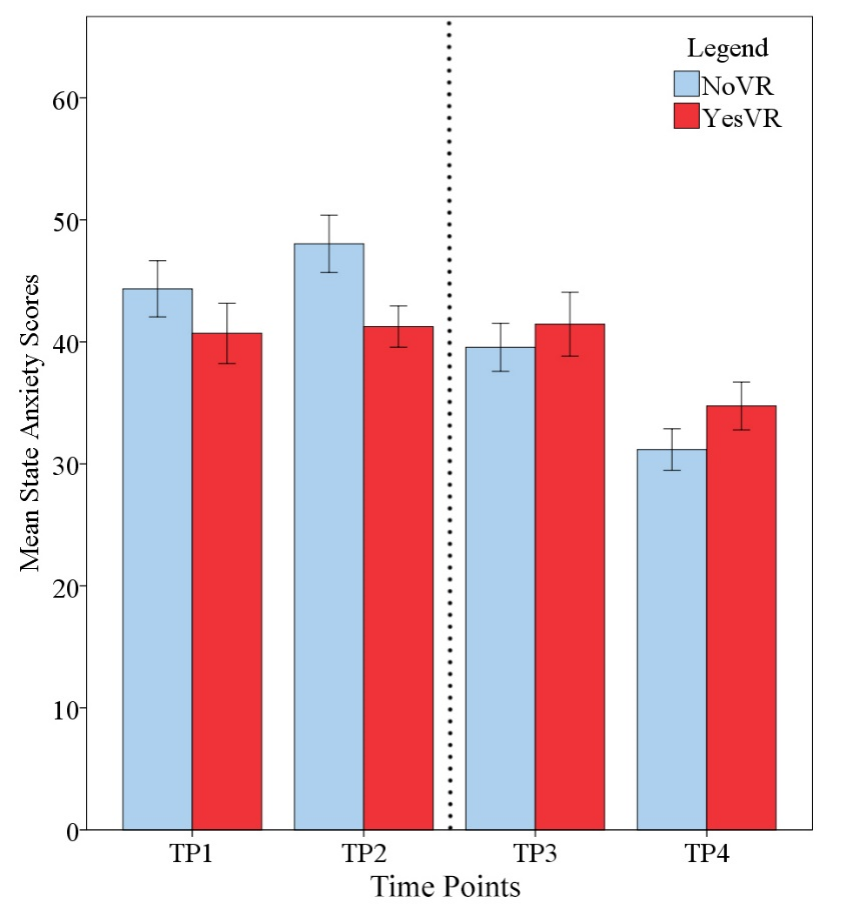
Student state anxiety at all time points (TPs); error bars represent standard error. The dotted line represents the time when the Objective Structured Clinical Examination (OSCE) took place. NoVR: subjects not exposed to the virtual reality simulation; YesVR: subjects exposed to the virtual reality simulation.

**Figure 4:**
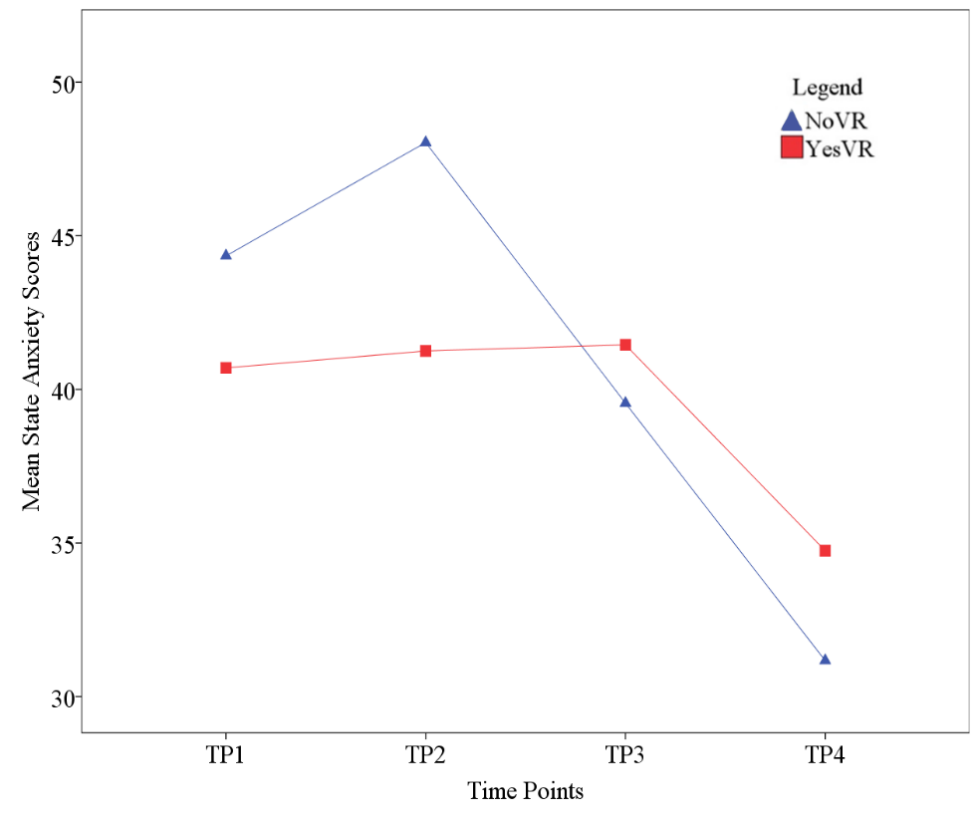
Student state anxiety at all time points (TPs). NoVR: subjects not exposed to the virtual reality simulation; YesVR: subjects exposed to the virtual reality simulation.

Figures 3 and 4 shows student state anxiety across time. The results of the 2 × 4 mixed ANOVA showed there was no significant main effect for phase (*F*_1,47_=0.276, *P*=.60, *ηp*^2^=0.006) on state anxiety scores, with NoVR (mean 40.78, SD 12.82) and YesVR (mean 39.54, SD 10.04) performing similarly overall. However, there was a significant difference in state anxiety scores between phases at TP2, with NoVR showing greater anxiety scores (mean 48.03, SD 12.67) than YesVR (mean 41.25, SD 7.54) (*t*_46.19_=2.34, *P*=.02, Cohen *d*=0.65, *ηp*^2^=0.105). The mean difference was 6.78 units (95% CI 0.96-12.61). Cronbach α values for the participant samples at TP2 were .95 and .87 for NoVR and YesVR groups, respectively.

**Table 1:** Student census data.

Year	Total class size (students), N	Aged 18-23 years, n (%)	Aged 24-29 years, n (%)	Aged 30-34 years, n (%)	Aged 35-39 years, n (%)	Identified as female, n (%)	Identified as male, n (%)
2017	123	13 (10.5)	106 (86.2)	3 (2.4)	1 (0.8)	109 (88.6)	14 (11.4)
2018	121	20 (16.5)	88 (72.7)	12 (9.9)	1 (0.8)	110 (90.9)	11 (9.1)

**Table 2:** Statistical analysis results.

Variables		Phase 1 (NoVR^a^), mean (SD)		Phase 2 (YesVR^b^), mean (SD)		*df*		ANOVA^c^						*t* test			*P* value (Cohen *d*^d^)		
								*F* test		*P* value		Partial *η*^2^							
**STAI^e^(Form Y-1) score at each time point (TP)**																			
	TP1		44.34 (12.38)		40.70 (11.06)		47		—		—		—			1.06			.30
	TP2		48.03 (12.67)		41.25 (7.54)		46.19^f^		—		—		—			2.34			.02 (0.65)
	TP3		39.55 (10.60)		41.45 (11.69)		47		—		—		—			–0.59			.56
	TP4		31.17 (9.17)		34.75 (8.76)		47		—		—		—			–1.37			.18
STAI (Phase)		—		—		47		0.28		.60		0.006			—			—	
STAI (Time)		—		—		3		18.40		<.001		0.281			—			—	
STAI (Intercept)		—		—		3		4.12		.008		0.081			—			—	
**TAI^g^at each TP**																			
	TP1		41.59 (14.07)		41.89 (12.99)		46^h^		—		—		—			–0.77			.94
	TP2		42.72 (13.55)		41.21 (11.58)		46^h^		—		—		—			0.40			.69
	TP3		40.62 (13.62)		41.68 (11.48)		46^h^		—		—		—			–0.28			.78
	TP4		40.52 (14.40)		41.79 (12.50)		46^h^		—		—		—			–0.32			.75
TAI (Phase)		—		—		46^h^		0.01		.94		<0.001			—			—	
TAI (Time)		—		—		3		0.67		.57		0.014			—			—	
TAI (Intercept)		—		—		3		1.57		.20		0.033			—			—	
**ASE^i^at each TP**																			
	TP1		18.90 (2.43)		19.20 (3.12)		47		—		—		—			–0.38			.70
	TP2		18.93 (2.37)		19.45 (3.10)		47		—		—		—			–0.66			.51
	TP3		19.79 (2.64)		19.80 (2.93)		47		—		—		—			–0.01			.99
	TP4		20.03 (2.76)		20.55 (2.72)		47		—		—		—			–0.65			.52
ASE (Phase)		—		—		47		0.22		.64		0.005			—			—	
ASE (Time)		—		—		2.62^j^		8.98		<.001		0.160			—			—	
ASE (Intercept)		—		—		2.62^j^		0.40		.73		0.008			—			—	

^a^NoVR: subjects not exposed to the virtual reality simulation.

^b^YesVR: subjects exposed to the virtual reality simulation.

^c^ANOVA: analysis of variance.

^d^Cohen *d* effect size is only reported for time point 2 (TP2).

^e^STAI: State-Trait Anxiety Inventory.

^f^Levene’s Test for Equality of Variances found equal variances not assumed; thus, *df* was changed accordingly.

^g^TAI: Test Attitude Inventory.

^h^Phase 2 (YesVR) had 1 participant fail to complete TAI surveys.

^i^ASE: Academic Self-Efficacy Scale.

^j^Mauchly’s test found sphericity assumption violated; thus, *df* was corrected using Greenhouse-Geisser estimates of sphericity (ε=.88).

There was a significant effect for time on state anxiety scores (*F*_3,141_=18.40, *P*<.001, *ηp*^2^=0.281, *f*=0.6), with participants showing a rise-then-fall trend in mean state anxiety scores across the time points (TP1=42.86, TP2=45.27, TP3=40.33, and TP4=32.63). Pairwise comparisons that were corrected with Bonferroni *t* tests and CI adjustments showed a significant difference between TP1 (mean 42.86, SD 11.88) and TP4 (mean 32.63, SD 9.09) (*P*<.001), with a mean difference of 9.56 units (95% CI 5.12-14.00). There was also a significant difference between TP2 (mean 45.27, SD 11.29) and TP4 (*P*<.001), with a mean difference of 11.68 units (95% CI 7.30-16.06). Lastly, a significant difference was found between TP3 (mean 40.33, SD 10.98) and TP4 (*P*<.001), with a mean difference of 7.54 units (95% CI 2.94-12.14).

There was a significant interaction between time and phase in terms of state anxiety scores (*F*_3,141_=4.12, *P*=.008, *ηp*^2^=0.081, *f*=0.25). Descriptive statistics showed that NoVR participants showed greater state anxiety scores for TP1 (mean 44.34, SD 12.38) and TP2 (mean 48.03, SD 12.67) than did YesVR participants for TP1 (mean 40.70, SD 11.06) and TP2 (mean 41.25, SD 7.54). However, the opposite pattern occurred at TP3 and TP4 with NoVR participants, where they showed lower state anxiety scores (mean 39.55, SD 10.60, and mean 31.17, SD 9.17, respectively) than did YesVR participants (mean 41.45, SD 11.69, and mean 34.75, SD 8.76, respectively).

These results show that NoVR participants’ state anxiety had a rise-then-fall trend, peaking at the time point just before the OSCE. At that point, students who had access to the VR clinical simulation showed less anxiety than did the control students. The YesVR participants’ state anxiety showed no change in state anxiety scores from TP1 to TP3, before falling at TP4.

### Test Anxiety

**Figure 5:**
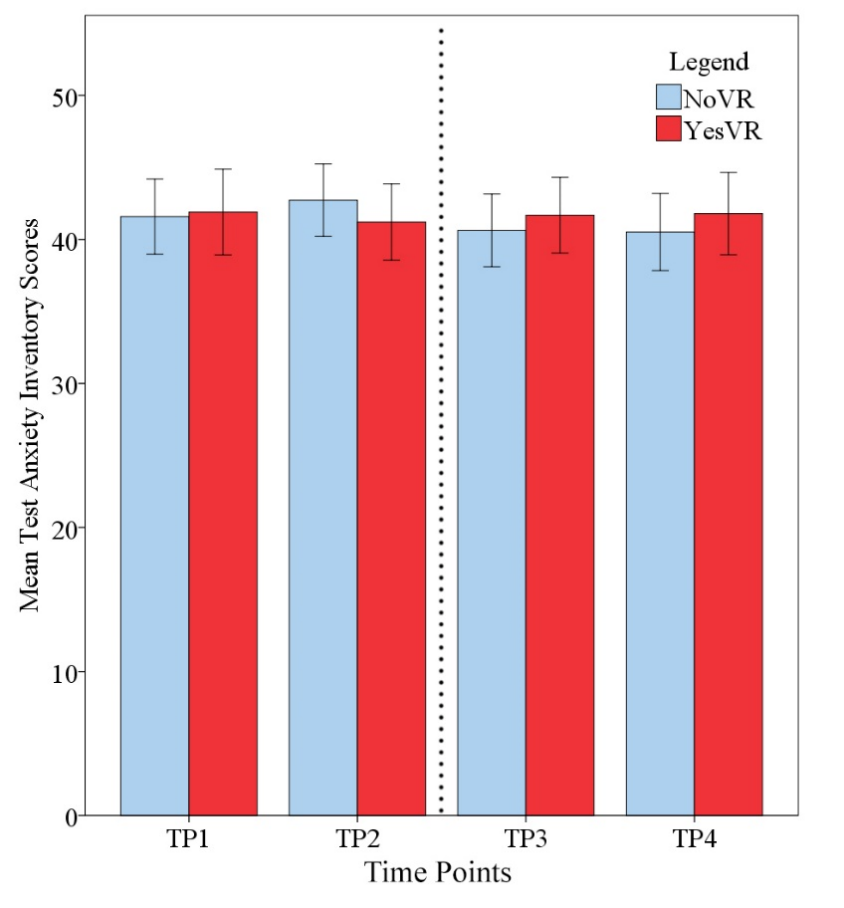
Student test anxiety at all time points (TPs); error bars represent standard error. The dotted line represents the time when the Objective Structured Clinical Examination (OSCE) took place. NoVR: subjects not exposed to the virtual reality simulation; YesVR: subjects exposed to the virtual reality simulation.

Figure 5 shows student test anxiety across time. The results of the 2 × 4 mixed ANOVA showed there was no significant main effect for phase (*F*_1,46_=0.005, *P*=.94, *ηp*^2^<0.001) on test anxiety inventory scores, with NoVR (mean 41.36, SD 13.76) and YesVR (mean 41.65, SD 11.74) participants performing similarly overall. There was no significant effect for time on test anxiety inventory scores (*F*_3,138_=0.674, *P*=.57, *ηp*^2^=0.014), with participants showing a similar level of scores across the time points (TP1=41.71, TP2=42.13, TP3=41.04, and TP4=41.02). There was no significant interaction between time and phase in terms of test anxiety inventory scores (*F*_3,138_=1.57, *P*=.20, *ηp*^2^=0.033).

These results show that the students’ test anxiety scores had remained relatively static throughout their participation in the OT program, whether they had access to VR or not.

### Academic Self-Efficacy

**Figure 6:**
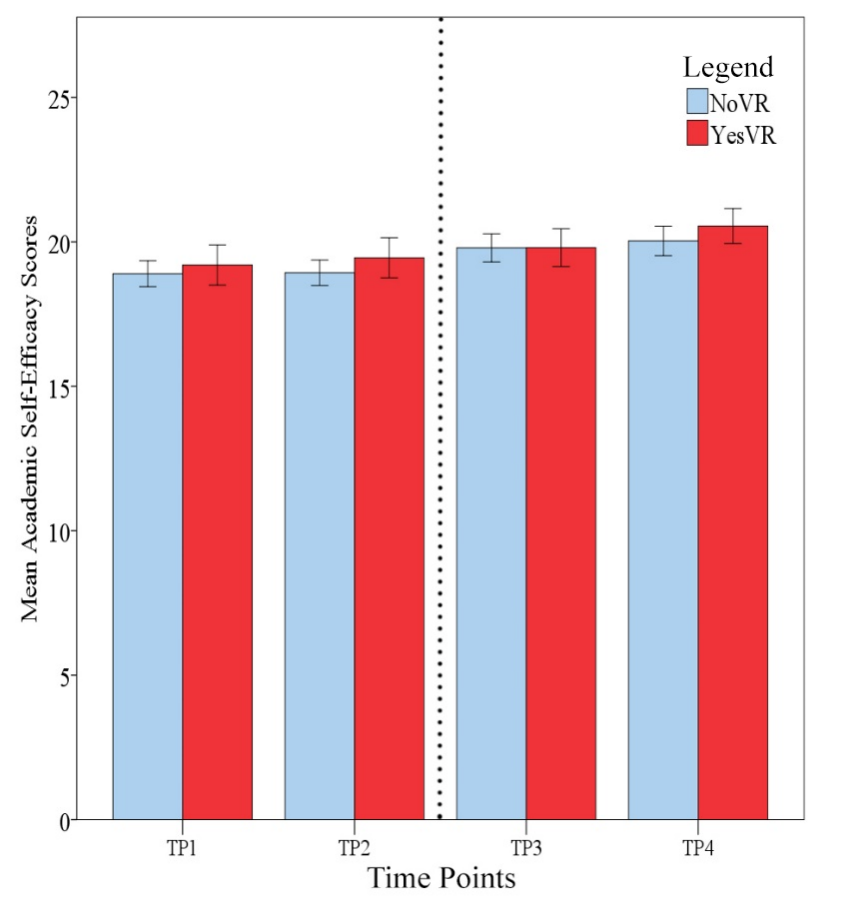
Student academic self-efficacy at all time points (TPs); error bars represent standard error. The dotted line represents the time when the Objective Structured Clinical Examination (OSCE) took place. NoVR: subjects not exposed to the virtual reality simulation; YesVR: subjects exposed to the virtual reality simulation.

Figure 6 shows student academic self-efficacy across time. The results of the 2 × 4 mixed ANOVA showed there was no significant main effect for phase (*F*_1,47_=0.217, *P*=.64, *ηp*^2^=0.005) on academic self-efficacy scores, with NoVR (mean 19.41, SD 2.57) and YesVR (mean 19.75, SD 2.96) participants performing similarly overall. Cronbach α values for the participant samples at TP2 were .61 and .74 for NoVR and YesVR groups, respectively.

Mauchly’s test indicated that the assumption of sphericity had been violated for the main effects of time: *χ*^2^_5_=13.32 (*P*=.02). Therefore, degrees of freedom were corrected using Greenhouse-Geisser estimates of sphericity (ε=.88). There was a significant effect for time on academic self-efficacy scores (*F*_2.62,123.32_=8.98, *P*<.001, *ηp*^2^=0.160, *f*=0.41), with participants showing an increase in mean academic self-efficacy scores across the time points (TP1=19.02, TP2=19.14, TP3=19.80, and TP4=20.24). Pairwise comparisons that were corrected with Bonferroni *t* tests and CI adjustments showed a significant difference between TP1 (mean 19.02, SD 2.70) and TP4 (mean 20.24, SD 2.73) (*P*=.001), with a mean difference of 1.24 units (95% CI 0.45-2.04). There was also a significant difference between TP2 (mean 19.14, SD 2.68) and TP4 (*P*=.002), with a mean difference of 1.10 units (95% CI 0.32-1.89).

There was no significant interaction between time and phase in terms of academic self-efficacy scores (*F*_2.62,123.32_=0.40, *P*=.73, *ηp*^2^=0.008). There was a significant moderate negative correlation between mean, total, academic self-efficacy (mean 19.14, SD 2.68) and mean, total, peak state anxiety scores at TP2 (mean 45.27, SD 11.30) (*r*=–.42, n=49, *P*=.003).

These results show that student academic self-efficacy had continually increased throughout their participation in the OT program, whether they had access to VR or not, yet it was inversely related to state anxiety at the peak anxiety time point.

### State Anxiety, Study Time, and Strategies

**Figure 7:**
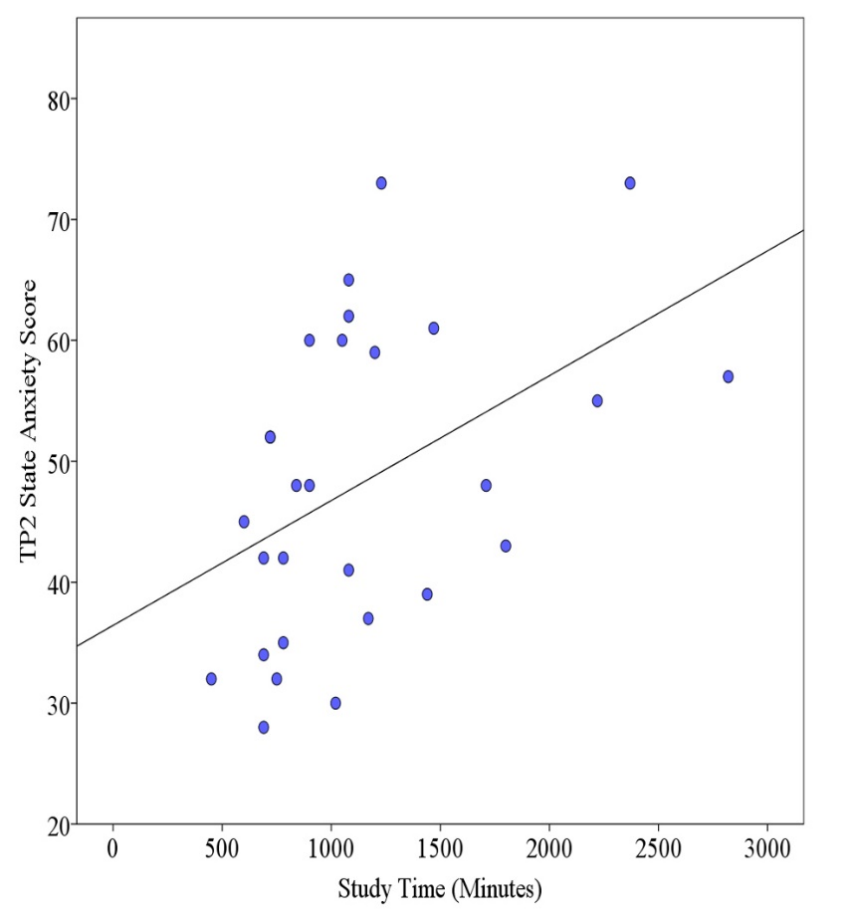
Correlation of student state anxiety and total study time at time point 2 (TP2) for subjects not exposed to the virtual reality simulation (NoVR). The line represents Pearson *r*=.46.

**Figure 8:**
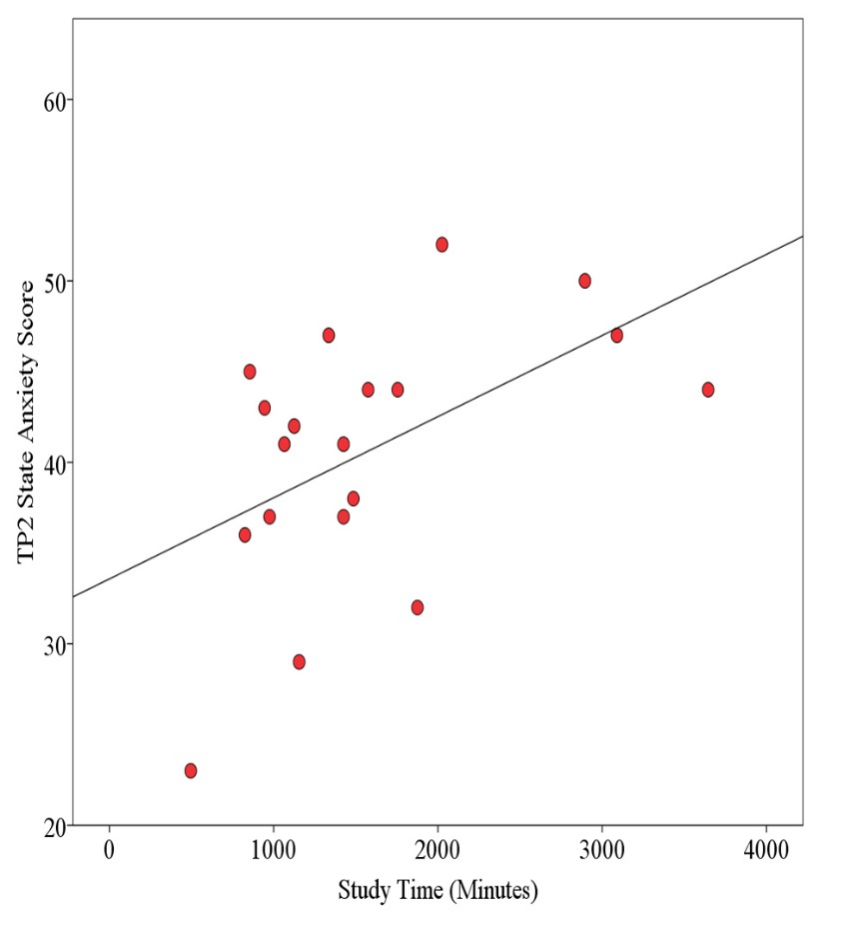
Correlation of student state anxiety and total study time at time point 2 (TP2) for subjects exposed to the virtual reality simulation (YesVR). The line represents Pearson *r*=.52.

Figures 7 and 8 show respective NoVR and YesVR phase correlations of student peak anxiety levels in relation to their total study times. For the NoVR participants, there was a significant moderate positive correlation between total study time and student state anxiety scores at TP2 (*r*=.46, n=28, *P*=.01). For the YesVR participants, there was a significant moderate positive correlation between total study time and student state anxiety scores at TP2 (*r*=.52, n=19, *P*=.02).

There was no significant difference in total study time per week between the NoVR group (mean 383 minutes/week, SD 191) and the YesVR group (mean 315 minutes/week, SD 166) (t_45_=1.27, *P*=.21, Cohen *d*=0.38). The top study strategies used by the NoVR group, as well as the mean percentages of time spent studying, were *hands-on group practice* (43.4%), *making and reviewing notes* (17.9%), *hands-on individual practice* (15.3%), and *reviewed e-class resources* (8.4%). The YesVR group used the same top study strategies in the same order: *hands-on group practice* (46.6%), *making and reviewing notes* (24.8%), *hands-on individual practice* (21.2%), and *reviewed e-class resources* (4.2%). The fifth-most common study strategy used by the NoVR group was *self-talk* (3.3%), while that of the YesVR group was *using the VR simulation* (1.1%).

The results show that excess time spent studying for clinical practical examinations was related to an increase in state anxiety. In summary, the top four study strategies used by the two groups were of the same type and were in the same order, yet the fifth-most common strategy for the NoVR group employed *self-talk* strategies, while the YesVR group used *VR simulation*.

### Interview and Previous Experience with VR Survey Responses

In short summary, common student statements regarding realism and presence of the VR simulation from our interviews and focus groups included the following:

Feels like I’m really taking the exam.

I almost freaked out when I saw the examiner in the room.

The virtual reality let me experience the clinical exam in advance, which helped a lot.

I’d love to work with more VR in the future.

Refer to Table 3 for student responses to the Previous Experience with VR Survey.

**Table 3:** Previous Experience with VR Survey responses.

Item	Response (N=20), n (%)
No previous experience.	12 (60)
Neither owned nor knew someone who owned an immersive VR (virtual reality) system.	17 (85)
Main interest for immersive VR was training and education.	13 (65)
Quality of graphics is the most important requirement to make VR simulation feel the most realistic.	19 (95)
Both quality of graphics and ease of use and interaction with the virtual environment are the most important requirements to make VR simulation feel the most realistic.	17 (85)
Prefer to use immersive VR for professional work and education.	19 (95)
Use VR for both professional work and education and learning new skills.	14 (70)
Believed VR would have at least moderate potential as an educational tool within the next 10 years.	10 (50)
Believed VR would be “the way of the future” in the next 10 years.	7 (35)

## Discussion

### Overview

This investigation shows evidence of immersive VR’s potential to reduce anxiety in OT students during their peak state anxiety time point as they prepared for an upcoming OSCE. However, this investigation did not fulfill the requirements of a rigorous randomized controlled trial. Thus, causation of immersive VR’s effectiveness for the reduction of anxiety in OT students in training cannot be inferred. Favorable demonstrations of immersive VR’s ability to reduce anxiety must consider that cognitive and affective responses, within the confines of a virtual world, could vary from those observed in the real world. The artificial nature of OSCEs are designated to act as simulations themselves, and despite their attempts to represent situations within the real world, they are not the real world [35]. Such can be stated for immersive VR simulation and its potential to optimize mental preparedness in medical students for the real world. Despite these remarks, previous research on immersive VR has demonstrated its capability for reducing state anxiety within the field of health sciences [20]. The results of this investigation reflected those trends.

### Main Findings

State anxiety in the OT students was found to be different between phases only at the time point located 1 week prior to the OSCE (ie, TP2), which encompassed the VR intervention for the purpose of *inhibitory learning* to occur. It cannot be ruled out that differences in participant characteristics, covariates, and coursework, or even the qualities of the students themselves who agreed to participate, may have been responsible for the difference in state anxiety observed. It also cannot be stated that student performance anxiety levels were reduced during their actual performance in the OSCE event itself, since no measures were taken at that time. Gaggioli and colleagues’ workplace stress report [20] showed a main effect for the reduction of anxiety in their VR experimental group, yet their study featured multiple VR sessions—eight treatment sessions—while the mode of this investigation featured only one. Although the state anxiety scores between groups was not significantly different at the first time point, the mean state anxiety being greater among the NoVR participants at that time may arguably have been the instigating difference for the second time point, despite test anxiety traits being similar for both groups.

First impressions of VR’s influence in this investigation would appear to be mixed. State anxiety levels were not found to be significantly reduced for the YesVR group across all time points. At the first time point, the VR intervention had not been applied; thus, a difference in state anxiety levels at this point would have indicated a confounding difference between the groups. For the second time point, after the VR intervention had been applied, the YesVR group demonstrated an absence of an increase in state anxiety. This may have been influenced by other potential confounders. Although this investigation was able to consider test anxiety traits, faculty and coursework consistency, and study strategies, these potential confounders were not rigorously controlled and may have been the prime determinants of state anxiety differences. At the third time point, both groups’ state anxiety levels returned to baseline levels, which were not significantly different from one another. This was after the OSCE had ended, yet the students were still working on other coursework, most likely subjecting them to different stressors than what was shown in the VR simulation. At the fourth time point, student anxiety levels were at their lowest, because this was when they were starting their next term with new coursework, when stressors have not yet manifested. These third and fourth time points were when the use of VR showed no retention of reduced state anxiety. These observations suggest that VR’s ability to reduce state anxiety is considerable when it matters most; a specific intervention is given 2 weeks before the peak anxiety time point for a specific stressor. However, VR’s ability to reduce state anxiety was not transferrable across different stressors, nor did it show a prolonged reduction in state anxiety over a longer period of time. The implication of these anxiety levels implies that OT departments, considering the incorporation of OSCEs for performance assessments, should expect students to experience increasing levels of state anxiety, especially when their OSCE appointments draw near. It is recommended that systems be implemented to mitigate this potential increase in state anxiety.

Test anxiety in OT students was not found to be different between phases or time points. Spielberger’s manual [26] states that the TAI was developed as a tool to measure test anxiety as a “situation-specific personality trait.” This could potentially be less sensitive to changes over time than Form Y-1 of the STAI, which is a measure that is sensitive to changes in state anxiety. Note that test-retest reliability of TAI scores for college and graduate students for time spans of 3 weeks and 2 weeks, respectively, have each been found to be strong (*r*=.80) [26]. Personality traits are expected to be stable and unlikely to change over time [36]. Despite this lack of difference, there are two points to consider:

1. Having no significant difference in test anxiety scores between phases at the first time point, prior to the VR simulation intervention, further establishes similarity between phases of OT students for test anxiety–specific personality traits, prior to their shown differences in state anxiety at TP2.
2. The implication of test anxiety–specific personality traits being the same across the time points means that student attitudes toward clinical practical exams are unlikely to change as they participate in an OT program.

If students suffer debilitating symptoms of test anxiety, this is unlikely to change as they continue with their OT program. OT programs are recommended to be equipped with separate and dedicated activities to mitigate test anxiety in students.

In order for immersive VR to have potential in reducing trait-based anxiety, it would require an established treatment protocol, similar to what Gaggioli and colleagues stated in their workplace stress report in 2014 [20]. Their treatment protocol followed the stress-management training program as established by Kaluza [37] and Meichenbaum [38], which consisted of 10 1-hour sessions in 5 weeks, administered by clinical psychologists [37-39]. A stress-management training program utilizing VR in this manner would be expected to reduce chronic workplace trait anxiety by 12%, greater than the results found when compared to a cognitive behavioral therapy control group [20].

This investigation unexpectedly found academic self-efficacy to gradually increase across the time points for both phases, despite an expected moderate inverse relationship being found at the peak state anxiety point. Greater academic self-efficacy being associated with lower state anxiety is congruent to Dobson’s report [8], which was reflected in this investigation’s peak anxiety time point. In a report featuring anxiety related to writing in graduate students, self-efficacy was found to have a large and inverse relationship with writing anxiety [40]. However, it has been stated in previous reports that low-to-moderate levels of emotionality (ie, affective physiological reactions to anxiety) may actually enhance a student’s performance, while excessive levels may cause a reduction in performance [41,42]. The peak state anxiety levels in the OT students within this investigation were not strong enough to show a noticeable reduction in their academic self-efficacy scores, possibly due to extraneous variables, such as mental resiliency and previous experience. Enjoyment in the learning material and student pride have been found to have positive associations for self-efficacy [43]. Future research that compares OT students’ peak state anxiety levels and their actual OSCE performance scores would allow further conclusions to be made for academic self-efficacy, state anxiety, and performance relationships.

There is a conceivable argument to be made for the necessity of students to endure anxiety symptoms as they progress through OT programs. By overcoming situations that induce anxiety, it is arguable that students will learn necessary coping skills to apply in practice for the real world. However, this investigation showed no difference in the rate of academic self-efficacy development between the YesVR and NoVR groups of OT students, despite their significant difference in peak state anxiety scores at TP2. Thus, it is presumed that OT students will not have their academic self-efficacy development compromised when VR interventions significantly reduce state anxiety levels. VR interventions that are designed to reduce state anxiety do not result in OT students missing out on academic self-efficacy development.

For the association between total study time and peak state anxiety, it is to be assumed that greater time spent in preparation for an upcoming OSCE is equivalent to students placing greater amounts of perceived importance onto the successful outcome of the evaluation. Students who appraise exams with high importance are associated with increased state test anxiety levels before the exam, which results in higher anxiety levels after the exam [44]. This association could also mean that facilitative aspects of anxiety may have compelled the students to spend greater amounts of time in preparation for the OSCE.

Based on participant responses from the Previous Experience with VR Survey, it appears that views regarding the adoption of VR simulation into OT programs are favorable, especially for use in professional work and education. The majority of students stated that the quality of graphics was the most important consideration for achieving realistic virtual environments, while also stating that ease of use and interaction were also important. However, there were other important factors, besides graphics, which may be just as vital in order to achieve a higher sense of fidelity. Although the speech-recognition system allowed the use of natural language to create the clinical interview experience, there were no measures of participant frustration or concern whenever they asked a question that the virtual patient failed to understand. Although the participants were instructed to reword questions or change the subject when encountering such events, these limitations in speech-recognition software may have negatively impacted the user’s sense of immersion, potentially impacting the system’s anxiety-reduction capability. Future designs are recommended to include a measure of the amount of virtual patient communicative misunderstandings and user immersion levels, determining if these variables are related.

For the verbal communication factors, there were limitations that may have impacted the user experience. For example, the avatars in this investigation all spoke with the same tone. They did not change their tone of voice to signify differing levels of severity to their condition. Differing tones of voice could have been used to communicate various types of emotion, which may have added to a user’s perceived level of fidelity. Depending on the choice of words a virtual patient uses, their messages could have negative or positive connotations. This investigation did not feature the changing of a virtual patient’s spoken words to influence negative or positive connotations. For example, a virtual patient could have said either “I hurt my shoulder” or “I ruined my shoulder” to imply different messages. Future virtual patient dialogue would benefit from these verbal communication factors being considered.

For the nonverbal communication factors, the virtual standardized patients were programmed to maintain eye contact with the user throughout the simulation, yet this extent of nonverbal communication could be improved upon with developed posture, gesture, and facial expression. Kinesics, such as posture, gesture, and facial expression, are encouraged to be implemented into virtual avatars to optimize the quality of communicative experiences [45]. The communicative properties of an avatar’s eyes (ie, oculesics), such as gaze, pupil dilation, and eyelid movements, are considered to have a major impact on a user’s perceived sense of realism [45]. Based on Steptoe’s report [45], varying these oculesics parameters in virtual standardized patients to match those of varying personality types or truth and deception responses may result in avatars that students may perceive to be socially real. A virtual environment that simulates having an interview with a patient, based mainly on social communication interactions, allows users to establish a sense of what is expected in the real world. Incorporating oculesics properties into virtual patients may instill a greater sense of immersion for the user to enhance their communicative experience.

### Strengths and Limitations

This investigation is potentially the first to implement an immersive VR intervention within the discipline of OT for the reduction of state anxiety in students preparing for an OSCE. This investigation aimed to minimize researcher bias by having minimal contact with the participants. The primary outcome measures for state and test anxiety, in addition to academic self-efficacy, were taken by established theory-based tools. This investigation was supplemented by secondary outcome measures, including student total study time, study strategies, and previous experience with immersive VR, which were deduced from the primary measures.

However, this investigation had some limitations. Measures of student performance anxiety were not taken during the actual OSCE event, which did not allow inferences to be made about immersive VR’s effectiveness for that specific occasion. There were no follow-up measures taken, such as during the students’ next year of preparation for their second OSCE, to determine whether immersive VR had an effect on long-term memory development for *inhibitory learning*. Physiological stress markers, such as cortisol levels in the blood, saliva, and urine as well as heart rate, were not measured to determine possible changes in affective anxiety components. The sample size of this investigation was satisfied for within-subject measures, but the sample size would need to be increased for establishing greater confidence in the between-subject measures. This investigation was unable to check for student covariates between the phases and did not perform the rigors of a randomized controlled trial. The total cost for the software, hardware, and development of the simulation itself was estimated to be over US $50,000, which may discourage the adoption of such a system in other OT facilities. It is important to note that technological improvements in immersive VR hardware and software development are becoming increasingly efficient, resulting in an increase of accessibility to this platform.

### Future Recommendations

In addition to implementing design changes for the rectification of limitations as stated in this investigation, future designs may consider the use of general or workplace-based self-efficacy questionnaires, establishing students’ perceived levels of competency for the professional world. A comparison of immersive VR’s performance, developed with improved artificial intelligence for interview skills training, evaluated with formal clinical performance assessments, could be implemented to establish students’ levels of performance for the professional world. Virtual standardized patients could also be developed to have unique traits, allowing for students to train for scenarios that would otherwise be difficult or possibly dangerous.

### Conclusions

This investigation shows evidence of immersive VR’s capability to reduce anxiety in OT students who communicated with virtual standardized patients using natural language. Although test anxiety potentially leads to worry cognitions, which can disrupt students’ attention, this investigation showed that academic self-efficacy continually increased in health science students as they persevered in their program. A combination of optimal study strategies and immersive VR simulation for the reduction of anxiety in health science students preparing for clinical practical exams can lead to a future of positive mental health change from the virtual to the real world. Will your next clinical interview take place in the virtual world?

## Appendix

Multimedia Appendix 1 Survey regarding previous experience with virtual reality technology.

Multimedia Appendix 2 Objective Structured Clinical Examination (OSCE) virtual reality avatar voice-recognition script (short version).

## Abbreviations

ANOVA: analysis of variance
ASE: Academic Self-Efficacy Scale
CPU: central processing unit
ηp_2_: partial η_2_
GPU: graphics processing unit
IPAT: Institute of Personality and Ability Testing
NoVR: subjects not exposed to the virtual reality simulation
OSCE: Objective Structured Clinical Examination
OT: occupational therapy
S-Anxiety: State Anxiety
STAI: State-Trait Anxiety Inventory
TAI: Test Attitude (Anxiety) Inventory
T-Anxiety: Trait Anxiety
TAS: Test Anxiety Scale
TLEF: Teaching and Learning Enhancement Fund
TMAS: Taylor Manifest Anxiety Scale
TP: time point
VR: virtual reality
VRET: virtual reality exposure therapy
WEQ: Worry Emotionality Questionnaire
YesVR: subjects exposed to the virtual reality simulation
