# Supplementary material for: Immersive Virtual Reality for the Reduction of State Anxiety in Clinical Interview Exams: Prospective Cohort Study
Source: JMIR Serious Games. 2020 Jul 9;8(3):e18313. doi: 10.2196/18313 (PMC7381040; doi:10.2196/18313)
Supplement: Multimedia Appendix 1 [file games_v8i3e18313_app1.docx]

## **Multimedia Appendix 1**

Previous Experience with Virtual Reality Technology

**Virtual Reality (Definition):** An artificial environment or simulation which is experienced through sensory stimuli (e.g., as sights and sounds) provided by a computer and in which one's actions partially determine what happens in the environment” (Merriam Webster, 2017).

**Instructions:** Please circle a letter-option to answer each question below:

1. Aside from the OSCE Preparation VR sessions, have you tried the latest VR headsets such as the Oculus Rift, HTC Vive, Playstation VR, Samsung Gear VR or Google Cardboard?
2. Never tried
3. Once
4. A few times
5. Regularly

#### Do you own any of the latest VR headsets or apps for systems such as the Oculus Rift, HTC Vive, Playstation VR, Samsung Gear VR or Google Cardboard?

1. No, not even my friends/coworkers do
2. No, but one or more of my friends/coworkers do
3. Yes, I own one
4. Yes, I own more than one

#### My interest in virtual reality includes (may circle more than one):

1. Entertainment and gaming
2. Touring and shopping
3. Training and education
4. No interest
5. Other (please specify):
6. Which of the following is the most important for making a virtual environment feel the most realistic (may circle more than one):
7. Quality of the graphics
8. Relatable content
9. Ease of use and interaction with the environment
10. Music and sound effects
11. Other (please specify):

#### What would you like to be doing in virtual reality? (may circle more than one)

1. Professional work/education
2. Entertainment and gaming
3. Arts / design
4. Learning a new skill
5. Other (please specify):

#### Between now and 10-years from now, how much potential do you think virtual reality has as an educational tool?

1. None
2. Minor
3. Moderate
4. Way of the future
5. Other (please specify) :
