# Supplementary material for: Immersive Virtual Reality for the Reduction of State Anxiety in Clinical Interview Exams: Prospective Cohort Study
Source: JMIR Serious Games. 2020 Jul 9;8(3):e18313. doi: 10.2196/18313 (PMC7381040; doi:10.2196/18313)
Supplement: Multimedia Appendix 2 [file games_v8i3e18313_app2.docx]

## **OSCE VR Avatar Voice Recognition Script (Short Version)**

| **Topic** | **User Questions/Statements that VR is Programmed to Respond to:** |
| --- | --- |
| Greeting | Greetings |
|  | Hello |
| Greeting: Question | Hi, how are ya? |
|  | How are you? |
| Introduction | What is your name? |
| Consent | Are you all right if we do a quick interview? |
|  | Are you okay if we do an interview together? |
| Purpose | Do you know why you are here today? |
|  | What brings you here today? |
| Injury: When | Approximately how long ago did you get hurt? |
|  | How long ago did you get injured? |
| Injury: How | Can you tell me how you injured yourself? |
|  | How did the injury occur? |
|  | How did this happen? |
|  | How did you get hurt? |
|  | How did you hurt yourself? |
| Injury: Where | Heard you had an injury, where does it hurt? |
| Pain: Now | Are you currently in pain? |
|  | Are you in pain right now? |
| Pain: Type | Does the pain radiate? |
|  | What kind of pain is it? |
| Pain: When | Does it hurt at night? |
|  | Does it hurt in the morning? |
|  | When does it hurt the most? |
| Pain: Severity | Are you in a lot of pain? |
|  | How badly does it hurt right now? |
|  | How much does it hurt? |
|  | On a scale of 1-10 how bad is the pain? |
| Medication: Use | Are you currently taking any medications? |
| Medication: Adherence | Do you have any difficulties with your medications? |
|  | Do you have any trouble managing your medications? |
|  | How many medications are you taking? |
| Past Medical History | Are there any medical conditions that I need to know about? |
|  | Do you have any concerns other than your shoulder injury? |
|  | Do you have any other conditions that may be relevant for me to know? |
|  | Do you have any other health concerns? |
| OT Knowledge | Are you familiar with what occupational therapists do? |
|  | Have you heard of occupational therapy before? |
| Mood: General | How do you feel your emotional health is? |
|  | What's your mood like today? |
| Mood Yes/No | Are you more angry or irritable since your injury? |
|  | Are you more prone to anger or sadness now? |
|  | Do you find the pain affects your mood at all? |
|  | Do you have any concerns with your mood or how you have been feeling lately? |
|  | Do you think that you are more prone to irritability or frustration now? |
|  | Do you think your mood has been affected by the injury? |
|  | Has the pain affected your mood at all? |
|  | Has your mood changed? |
| Fatigue | Do you feel as energetic as before? |
|  | Do you feel that fatigue is an issue for you? |
|  | Do you feel tired or fatigued more often than usual? |
|  | Do you find that you tire more easily now? |
|  | Have you noticed a change in your energy levels? |
| Fatigue Difficulties | Do you feel tired after a specific activity? |
|  | Do you find working really fatiguing? |
| Pain: Cognition | Can you focus or concentrate on tasks like you were able to before? |
|  | Does the pain affect your concentration? |
| Pain: Sleep Disturbances | Are you having troubles falling asleep at night? |
|  | Does it hurt when you go to sleep? |
|  | Have you been sleeping ok? |
| Occupation: General | Has the injury impacted your ability to do anything? |
|  | Has your injury affected your ability to do what is important to you? |
| Self-Care | Are you able to do things like dress yourself or brush your teeth? |
|  | Are you able to put on clothes and shoes yourself? |
| Productivity: What | Are you engaged in any volunteer work? |
|  | Are you engaged in paid employment? |
|  | Are you in school? |
|  | Are you still working? |
|  | What do you do for a living? |
|  | What kind of work do you do? |
|  | Where do you work? |
| Productivity: Work Difficulties | Are there any aspects of your work that you find difficult? |
|  | Do you find it hard to do your job because of your injuries? |
|  | Do you have any current difficulties completing your work? |
|  | How are you finding work? |
|  | Is there any parts of your work that it's tough for you to do right now? |
| Productivity: Household (Who) | Who does the cleaning around the house? |
| Productivity: Household Yes/No | Are there things around the house that you are unable to do now? |
|  | Are you able to use the vacuum or dust? |
|  | Are you still able to manage household chores and activities? |
|  | Are you still able to manage your household tasks? |
|  | Can you lift heavy items, like garbage bags or groceries? |
|  | Do you have any concerns with household chores? |
| Productivity: Household Difficulties | Are there any concerns with finance management? |
|  | Has your injury made it difficult for you to pay your bills? |
| Productivity: Finances (Who) | Do you pay your own bills at home, or does someone else take care of that? |
|  | Who manages the money at home? |
|  | Who takes care of the finances? |
| Productivity: Transportation (How) | Are you able to get to where you need to go? |
|  | Do you drive or do you take the bus? |
|  | How do you get around? |
| Productivity: Transportation Difficulties | Do you find it difficult to navigate the buses? |
|  | Do you have any concerns with driving? |
|  | Do you have anyone who is able to drive you around? |
|  | Is there anyone else who is able to help you get to where you need to go? |
|  | Have you noticed any difficulties driving? |
| Leisure: What | In your spare time, what do you do to occupy the time? |
|  | Tell me about some of your hobbies? |
|  | What do you do for fun? |
| Leisure: Difficulties | Do you find that you are able to do your leisure activities? |
| Spirituality: Yes/No | Can you tell me a bit about what spirituality means to you? |
|  | Do you have any religious practices? |
|  | Is spirituality an important part of your life? |
|  | Would you say that you are affiliated with any religions? |
| Spirituality: General | Can you tell me a bit about what spirituality means to you? |
|  | What do you hope to achieve in life? |
|  | What do you value in life? |
| Physical: Describe | Can you describe what your home looks like? |
|  | How many stairs do you have in your home? |
|  | Is your bathroom and bedroom on the same floor? |
|  | What kind of a house do you live in? |
| Physical: Reaching | Are there any high places that you need to reach? |
|  | Do you find it difficult to reach certain things in your home? |
| Social: Yes/No | Can anyone in your family help you with that? |
|  | Can you get help if you needed it? |
|  | Do you feel comfortable asking someone for help if you need? |
|  | Do you have supports to help you? |
| Social: General | Are your family or friends available to help you with the cooking or cleaning? |
|  | Are your friends and family able to help you if you need? |
|  | Who would you say are your supports? |
| Intimacy | Are you sexually involved with a partner now? |
|  | Do you have any problems with sex or intimacy? |
|  | Have you been sexually involved with a partner in the past? |
|  | How has your injury or disability affected your sexuality? |
| Intimacy: Consent | People may experience sexual concerns related to their illness or disability so I am going to ask you a few questions around that now |
|  | Sometimes an injury or disability may impact people's ability to have sexual intercourse. Would it be okay for me to ask you some questions on that subject? |
| Movement Screening related | *The verbs in the questions below can be interchanged with appropriate words such as “raise, reach up with, reach down with, reach back with, rotate(ing) forward/backward”* |
| Movement: Ask Anatomical | Can you abduct your shoulder? |
|  | Can you extend your shoulder? |
|  | Can you externally rotate your shoulder? |
|  | Can you flex your shoulder? |
|  | Can you horizontally abduct your shoulder? |
|  | Can you horizontally adduct your shoulder? |
|  | Can you internally rotate your shoulder? |
| Injury: Confirm Location | Are you having trouble moving your arm towards the ceiling? |
|  | Are you having trouble moving your shoulder? |
|  | Is it hard to lift your arm above your head? |
